# Exploring the Relationship Between Visual Aesthetics and Social Commerce Through Visual Information Adoption Unimodel

**DOI:** 10.3389/fpsyg.2021.700180

**Published:** 2021-09-06

**Authors:** Yongzhong Yang, Yunyan Tang, Yu Zhang, Ruo Yang

**Affiliations:** Business Administration, Business School, Sichuan University, Chengdu, China

**Keywords:** social commerce, visual aesthetics, visual communication, micro-celebrity, self-construal, visual information adoption unimodel

## Abstract

The visual revolution and attention economy of the digital world have put visual aesthetic communication into the primary position of social media marketing. However, this phenomenon remains underexplored within social commerce research. This study thus develops a visual information adoption unimodel (VIAUM), to explore the relationship between visual aesthetics and social commerce intentions. Users with social commerce experience are invited to complete our online survey, and 321 valid data are collected. The results reveal that visual aesthetics has direct and indirect (*via* perceived usefulness) effects on the social commerce intention of users. Besides, interdependent self-construal (InterSC) strengthens the direct effect between visual aesthetics and social commerce intention. In contrast, independent self-construal weakens the mediation effects of perceived usefulness. This study is among the first attempts to empirically examine the intervening mechanism and boundary conditions between the visual aesthetics of self-presentation of micro-celebrity and the social commerce intention of consumers.

## Introduction

With the advancement of digital technology and the ubiquity of social networking, social commerce has emerged as a trendy business model of e-commerce (Liang and Turban, 2011; Chen and Shen, 2015; Busalim and Hussin, 2016). By integrating social media tools and web 2.0 into e-commerce, social commerce enables users to share commercial-related information with/from peer users online (Chen and Shen, 2015; Busalim and Hussin, 2016; Han et al., 2018), and thus is also known as “referral economy” (Wang and Zhang, 2012). Recently, the prevalence of visual-oriented social media platforms, such as Instagram, Facebook, and TikTok, has made visual information the dominant form of online communication over verbal information (Wang and Zhang, 2012; Schroeder, 2013). Meanwhile, the visual-mediated environment also raises attention distraction and choice overload challenges for social commerce business (Townsend and Kahn, 2014; Kusumasondjaja, 2019). In such a condition, catching “eyeballs” and strategically presenting visual information become the keys to the success of social commerce (Schroeder, 2004; García-Rapp, 2017; Aljukhadar et al., 2020).

Individuals usually adopt a set of attention-getting techniques for self-presentation and strategic communication (Marwick, 2015; Djafarova and Trofimenko, 2019), among which visual aesthetics is of top priority (Kusumasondjaja, 2019). Visual aesthetics refers to the aesthetic style of self-presentations embedded in visual communication strategies of the presenter and the extent to which other users perceive these presentations to be visually appealing and aesthetically impressive (Sanchez-Franco and Rondan-Cataluña, 2010; Liu et al., 2013). The significant role of visual aesthetics of websites has been widely discussed in the e-commerce context (Harris and Goode, 2010; Liu et al., 2013; Lorenzo-Romero et al., 2013). However, unlike traditional e-commerce that largely relies on system features to enhance shopping efficiency, social commerce prioritizes the power of user-generated content to drive commercial performance (Chen and Shen, 2015; Busalim and Hussin, 2016; Han et al., 2018). Since individuals control these social media contents, the visual aesthetics of the content results from the deliberate self-presentation of the individuals (Djafarova and Trofimenko, 2019). As such, it is the visual aesthetics of self-presentation of individuals rather than that of the web system that should be underscored in a social commerce context. Furthermore, in the lens of social information, visual aesthetics is a “bearer of meaning” that embodies the engaging lifestyle and unique tastes of presenters (Marwick, 2015; Brydges and Sjöholm, 2019; Leaver et al., 2020, p. 55). In other words, visual aesthetics can serve as a critical visual communication strategy that attracts attention and conveys meaningful information (Schroeder, 2011; Marwick, 2015; Aljukhadar et al., 2020; Leaver et al., 2020). However, despite its significance, little empirical research has examined how visual aesthetics works on the decision-making of consumers, especially in a social commerce context. This study, thus, aims to fill this gap by investigating the relationship between visual aesthetic presentations and social commerce intentions.

Based on the existing literature on visual heuristics, information adoption, and the single-route persuasion model, this study develops a visual information adoption unimodel (VIAUM), specifying visual aesthetics of the visual aesthetics of self-presentation of micro-celebrity as the antecedent, perceived usefulness as the mediator, and self-construal as the boundary condition, to understand how visual aesthetics works on social commerce intention. The present research expects to contribute the literature from three perspectives. First, focusing on the visual presentations of micro-celebrities, we demonstrate that a visual aesthetic self-presentation strategy is effective in social commerce marketing. Micro-celebrities are online users who achieve status as opinion leaders and can affect other behaviors and decision-making of users due to their attractive and influential posts (Djafarova and Trofimenko, 2019; Jin et al., 2019; Al-Emadi and Ben Yahia, 2020). Accordingly, focusing on user-generated from micro-celebrities provides us a better perspective to understand the impact of social information on social commerce platforms. Besides, existing literature suggests that the rise of micro-celebrities is primarily due to the increasing popularity in the practice of visual self-presentation (Schroeder, 2011; Marwick, 2015; Djafarova and Trofimenko, 2019). Micro-celebrities usually conduct various elaborate and aesthetic appealing tactics for self-presentations to accumulate their follower base and get commercial rewards from the “advertorials” of product and service (Abidin, 2016; Pedroni, 2017; Giles and Edwards, 2018). Therefore, working on visual aesthetic strategies of micro-celebrities is conducive to acquiring in-depth knowledge about strategic visual communication in social commerce.

Second, this study introduces perceived usefulness as the mediator between visual aesthetics and social commerce intention. Past research focuses mainly on physical attractiveness as a source characteristic for the visual effects of micro-celebrities (Chu and Kamal, 2008; Lee and Watkins, 2016; Sokolova and Kefi, 2020). From this perspective, visual aesthetics was thus seen as a content-irrelevant peripheral cue for persuasion (Sussman and Siegal, 2003). Besides, regarding the impact on consumer behaviors, most of these studies stressed the emotional functions of visual aesthetics of micro-celebrities, such as parasocial interaction (Lee and Watkins, 2016; Sokolova and Kefi, 2020) and attachment (Ki et al., 2020). However, the visual presentation is usually the composite of images and words or spoken languages, making it hard to distinguish between the central argument and source information (Couper et al., 2007). For instance, a visual presentation showcasing micro-celebrity wearing garments or experiencing certain services can provide aesthetic experience in parallel with product/service-related information. Accordingly, inconsistent with the visual communication literature (Schroeder, 2004; Harris, 2006; Couper et al., 2007; Simmonds et al., 2020), this study regards visual aesthetics as a source of information that combines aesthetic experience with valuable messages. In the established persuasion theories, the perceived usefulness of the information is a determinant factor in information adoption behaviors of receivers (Sussman and Siegal, 2003; Mudambi and Schuff, 2010). Therefore, our research examines the utilitarian function of visual aesthetics on social commerce intention *via* an intervening mechanism of perceived usefulness.

Finally, we tried to understand the effectiveness of visual aesthetic communication by relating self-construal to the depth of processing and capacity regarding visual information. As human behavior is formulated by the complex interaction of external incentives and individual factors, visual information adoption behaviors would vary between recipients (Singelis, 1994; Tsai, 2007; Braun et al., 2013; Hu et al., 2016). Self-construal is a remarkable discriminator influencing consumer responses to various marketing stimuli (Tsai, 2007; Hu et al., 2016; Aljukhadar et al., 2017; Haberstroh et al., 2018). It refers to the definition of self and structure of self-schema of an individual in relation to the surroundings (Markus and Kitayama, 1991; Ahluwalia, 2008; Aljukhadar et al., 2017). Individuals with independent self-construal (IndSC) view themselves as individuated entities distinct from others who value uniqueness and internal attributes. People with interdependent self-construal (InterSC), in contrast, hold a more socially embedded self-view that emphasizes connectedness, social contexts, and interpersonal relationships (Markus and Kitayama, 1991; Haberstroh et al., 2018). Reflecting the perceptions, evaluations, and behaviors of the individuals regarding the relationship of the self to the contexts (Markus and Kitayama, 1991), self-construal has been used to explain differences of the individuals in attention, perception, and interpretation of visual information (Cross et al., 2011; Han and Humphreys, 2016; Haberstroh et al., 2018). Hence, we examined the boundary condition of direct and indirect influences of visual aesthetics on social commerce intention by taking self-construal as the moderator.

## Theory and Hypotheses Development

### Theoretical Model

#### Visual Aesthetics as a Visual Heuristic

Recent literature on heuristic decision-making has put forward a new theoretical perspective for visual communication (Harris, 2006; Gigerenzer and Gaissmaier, 2011; Toepoel and Dillman, 2011; Townsend and Kahn, 2014; Berube, 2019). Heuristics is known as “efficient cognitive processes” or “mental shortcuts,” which involve the advantages of effort reduction and/or attribute substitution (Gigerenzer and Gaissmaier, 2011; Berube, 2019). Pieces of research point out that the human brain processes visual depiction profoundly faster than verbal and textual stimulus (Townsend and Kahn, 2014; Seifert and Chattaraman, 2020). Besides, visual presentations are verified to be more memorable and more directly connected to meaning than verbal depiction (Townsend and Kahn, 2014; Marwick, 2015; Jin and Ryu, 2019). In addition, following the principle of “bounded rationality” (Simon, 1955), several academics indicate a “less is more” effect of simple heuristics in which heuristics is more accurate than rational models (Wübben and Wangenheim, 2008; Gigerenzer and Gaissmaier, 2011; Saab and Botelho, 2020). Visual heuristics, thus, refers to methods that make use of salient visual cues for more accessible, quicker, and/or more accurate decision-making than complex approaches (e.g., the systematic mode in ELM and the central route in HSM, Kruglanski and Thompson, 1999; Gigerenzer and Gaissmaier, 2011; Saab and Botelho, 2020).

Studies suggest that heuristic strategies are particularly applicable to “large worlds” where the environment is complex, full of uncertainty, and quick decisions are needed (Wübben and Wangenheim, 2008; Gigerenzer and Gaissmaier, 2011). Based on the logic of cognitive saving, visual heuristics is also proved to be efficient in attention-seeking under the “ephemeral but omnipresent” interactive visual ecology (Harris, 2006; Couper et al., 2007; Marwick, 2015; Zulli, 2018). Social commerce involves a business environment of information saturation and content overload (Marwick, 2015; Kusumasondjaja, 2019), where cognitive limitations exist in terms of recognizing all relevant knowledge (Gigerenzer and Gaissmaier, 2011), and thus attentions become scarce and elusive resources to obtain (Zulli, 2018). Besides, Pallak et al. (1983) suggest that a visual-oriented environment encourages heuristic processing for information judgments. This tendency is also confirmed in recent social-media literature in which visual contents are found to be more welcoming than written texts among online users (Townsend and Kahn, 2014; Marwick, 2015; Kádeková and Holienčinová, 2018). In short, visual heuristics should be critical strategic communication tools in the context of social commerce.

The impression of visual aesthetics results from the innate visceral response of an individual that hardly requires cognitive effort to make a judgment (Lorenzo-Romero et al., 2013; Palmer and Peterson, 2016). Evidence shows that visual aesthetics can generate a first impression of a website (Lorenzo-Romero et al., 2013), or that of an individual (Stockemer and Praino, 2017), within 100 ms of exposure. Meanwhile, existing literature indicates that visual aesthetics generates a halo effect that increases the overall evaluation of the targets, known as “attractiveness premium” (Lucker et al., 1981; Palmer and Peterson, 2016). Past studies have confirmed visual aesthetics as a practical heuristic shortcut in influencing attitudes and behaviors of individuals, especially in “low information rationality” environments like political elections (Palmer and Peterson, 2016; Stockemer and Praino, 2017) and online markets (Phelan et al., 2011; Bhandari et al., 2019).

#### Visual Information Adoption Unimodel

The information adoption model (IAM, Sussman and Siegal, 2003) is an appropriate theoretical framework to explore the persuasive mechanism of visual information in social commerce environments (Chung et al., 2015; Erkan and Evans, 2016; Tapanainen et al., 2021). Information adoption model explains an informational influence process in which information adoption intentions of individuals are based on their perceived information usefulness influenced by information argument and source credibility (Sussman and Siegal, 2003). Peripheral cues, such as visual aesthetics, are treated as content-independent information from external sources whose persuasive power is believed to be less critical than argument messages when forming evaluations (Kruglanski and Thompson, 1999; Sussman and Siegal, 2003). Nevertheless, we suggest that IAM is not fully applicable to visual communication for the following reasons.

First, the original IAM is conducted in a text-based media where peripheral cues cannot reflect the message itself (Sussman and Siegal, 2003). In a visual-based context (e.g., social commerce); however, things may be complicated. Visual language is “the integration of words, images, and shapes into a single communication unit” (Horn, 1998, p. 8). Visual heuristics thus is seldom viewed in isolation from contents (Harris, 2006; Couper et al., 2007). For example, visual presentations of micro-celebrities usually include relevant product attributes in parallel with the showcase of themselves in the form of he/she who is wearing the products or experiencing the services (McQuarrie et al., 2013; Jin and Ryu, 2019). Therefore, peripheral cues from external sources can also be information-relevant. In some cases, visual aesthetics is even considered as the most essential “hardware” (i.e., central arguments) for a product or service (McFarlane and Samsioe, 2020). For instance, individuals may find a visual aesthetic presentation of a micro-celebrity as inspiring information that provides helpful aesthetic style advice such as outfitting matching and wearing effects (McCormick and Livett, 2012). Customers thus would rely on source attributes such as visual styles and physical attractiveness for the diagnostic evaluations of products or services (Yang et al., 2010; Schroeder, 2013; Schnurr et al., 2017). In other words, there is no absolute dichotomy between visual heuristics and message arguments as suggested in dual-route models (Kruglanski and Thompson, 1999).

For these reasons, we suggest that a unimodel of IAM may be more suitable for the present study. Unimodel is a single-route persuasion model developed upon lay epistemic theory (LET) that demonstrates the process of reasoning from evidence to conclusion depended on motivation and cognitive capacity (Kruglanski and Thompson, 1999). Unimodel regards both heuristics and message arguments as equivalent-functional evidence to make evaluative inferences, making no distinct processes of information types (Kruglanski and Thompson, 1999). Instead, it allows an in-depth and extensive discussion about the “cognitive responses to persuasion” rooted in motivation and cognitive abilities (Kruglanski and Thompson, 1999). In short, unimodel holds a fundamental belief that the motivation and cognitive abilities of individuals rather than the information type will differ the persuasive results.

In conclusion, by integrating visual heuristic theories (Harris, 2006; Gigerenzer and Gaissmaier, 2011; Berube, 2019), IAM (Sussman and Siegal, 2003), and persuasive unimodel (Kruglanski and Thompson, 1999), we construct a VIAUM in an effort to provide a new framework for understanding the visual communication in a social commerce environment. Primarily, we take visual aesthetics—a predominant visual heuristic strategy—as inferential evidence (information) that will work on information adoption behaviors of individuals through the intervening effect of perceived usefulness. Kruglanski and Thompson (1999) indicate that motivation and cognitive capacity of the individuals would exert different cognitive responses to persuasion and thus influences the judgment formation process. Self-construal is a self-related concept that reflects the differences of the individuals in motivation and cognitive styles (Cross et al., 2011; Liu et al., 2015; Cheek and Norem, 2017). As such, we assume that self-construal may play as a moderator in our proposed framework that will make a significant difference in persuasion effects. Moreover, visual aesthetics will exert preferential choices in addition to cognition and interpretations (Schroeder, 2004; Townsend and Kahn, 2014). Gigerenzer and Gaissmaier (2011) indicate that inferences and preferences of visual heuristics are usually embedded in identical cognitive processes. Thus, we infer that visual aesthetics will also drive a direct effect on information adoption due to a process of preferential choice (Gigerenzer and Gaissmaier, 2011; Townsend and Kahn, 2014). Besides, scholars suggest that rational trade-offs and capacity limitations will activate visual heuristics for decision-making (Gigerenzer and Gaissmaier, 2011), which implies a possible moderating effect of motivations and cognitive abilities between visual aesthetics and information adoption intentions.

**Figure 1:**
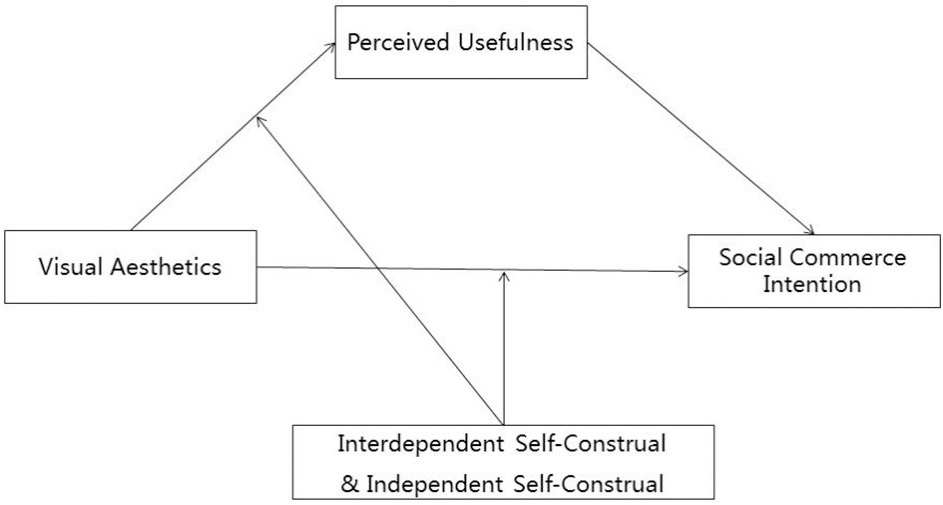
Visual information adoption unimodel (VIAUM) in the context of social commerce.

Moreover, individuals usually engage in information-seeking and obtaining activities for better purchasing decisions on social commerce platforms (Busalim and Hussin, 2016; Han et al., 2018). Social commerce involves receiving shopping information and products on social networking sites to support purchasing decisions (Hajli and Sims, 2015; Horng and Wu, 2020). Moreover, by applying commercial functions to social networking sites, social commerce also implies engaging in direct transactions on social commerce platforms (Hajli, 2013; Fu et al., 2019). In sum, referring to the willingness to accept the recommendation or engage in direct trading on the social commerce platform, social commerce intention is then used as a replacement of information adoption to fit in the context of social commerce. Our integrated theoretical model is thus presented in Figure 1.

### Hypotheses Development

#### Visual Aesthetics and Social Commerce Intention

In the practice of micro-celebrity, visual aesthetics usually refers to a kind of self-presentation strategy that involves a careful and elaborate aesthetic design of visual presentations (e.g., plog, vlog, and livestreaming) to attract attention, convey meanings as well as obtain commercial rewards (Schroeder, 2004; Marwick, 2015; Zulli, 2018; Brydges and Sjöholm, 2019; Jin and Ryu, 2019; Leaver et al., 2020). It encourages the use of aesthetic tactics (such as filters, Photoshop, and beauty devices) to share product/service-related information or embed commercial messages into the display of attractive appearances and aspirational visions of life (Schroeder, 2004, 2011; Brydges and Sjöholm, 2019; Jin and Ryu, 2019; Leaver et al., 2020). To be noticed, we consider the physical attractiveness of micro-celebrities as a component of their visual-presentation strategies instead of merely a preordained source attribute (as discussed in source credibility models). With this regard, the physical attractiveness indicates carefully crafted body efforts of micro-celebrities that thus embodied a taste or aesthetic style with a judgmental power (McQuarrie et al., 2013; Abidin, 2016; Jin and Ryu, 2019; Ki and Kim, 2019).

Past studies suggest that visual aesthetics of websites is the “decisive trigger” for social media engagement of consumers (Greussing and Boomgaarden, 2019). Visual aesthetics can contribute to the overall evaluations of interfaces (Phelan et al., 2011; Lorenzo-Romero et al., 2013; Greussing and Boomgaarden, 2019), impulsive purchase intentions (Liu et al., 2013; Park et al., 2015), and online purchase intentions (Schnurr et al., 2017). Meanwhile, literature on influencer marketing has suggested that the visual aesthetics of self-presentations are the main drivers to draw attention and the perquisites of users of being influential on the social media platforms for micro-celebrities (Marwick, 2015; Djafarova and Trofimenko, 2019; Ki and Kim, 2019). When consumers find self-presentation of a micro-celebrity visually attractive, they will follow him or her and exert approach behaviors (Haberstroh et al., 2018). Besides, past research indicates that attractive salespeople positively impact the products associated with them and thus increase purchase intention of consumers (Ahearne et al., 1999). Besides, as a type of visual heuristics, visual aesthetics will also activate shortcut judgment of consumers and thus prompt consumers to make quick and intuitive decisions, especially in an information-rich but uncertain social commerce environment (Gigerenzer and Gaissmaier, 2011; Wang and Zhang, 2012; Berube, 2019; Ishfaq et al., 2020). Hence, we predict that:

H1: visual aesthetics is positively related to social commerce intention.

#### The Mediating Role of Perceived Usefulness

Perceived usefulness refers to the extent to which individuals regard the information as beneficial for their tasks (Sussman and Siegal, 2003, p. 49). It is a critical information diagnostic indicator in the decision-making of people (Mudambi and Schuff, 2010). The relationship between aesthetics and usability has been confirmed in the field of information systems (Tractinsky et al., 2000; Sanchez-Franco and Rondan-Cataluña, 2010; Longstreet et al., 2021). Based on the “Gestalt” approach, theorists suggest that individuals intend to organize different elements of a visual presentation together to make an overall judgment as well as the associated inference from one another (Tonder and Van Spehar, 2013; Park et al., 2015; Bhandari et al., 2019). Consequently, a good impression of visual aesthetics will result in a favorable inference on other attributes of the whole visual presentation, such as the usability of websites (Sanchez-Franco and Rondan-Cataluña, 2010; Longstreet et al., 2021). Meanwhile, Coursaris and Van Osch (2016) and Wang et al. (2019) claim that visual aesthetics can reduce cognitive load and thus increase the efficiency and usability of the websites. A negative aesthetic impression toward design of websites, on the other hand, hinders the cognitive elaboration process of information due to a waste of cognitive resources and thus decreases the perceptions of usefulness (Coursaris and Van Osch, 2016; Greussing and Boomgaarden, 2019). There is relatively little examination of the utilitarian function of visual aesthetics in the field of influencer marketing. However, qualitative studies suggest that consumers did take micro-celebrities' visual aesthetics as practical style advice that provides them inspiration and information for better grips with products when considering a purchase (McCormick and Livett, 2012; Djafarova and Trofimenko, 2019).

Perceived usefulness is a critical predictor of the intentions of individuals to adopt certain information (Sussman and Siegal, 2003; Chung et al., 2015; Erkan and Evans, 2016; Tapanainen et al., 2021). For example, Chung et al. (2015) indicate that the perception of usefulness of UGCs affects adoption intentions of users for online travel information. Erkan and Evans (2016) prove that a positive e-WOM increases purchase intention of consumers through the perception of information usefulness. Tapanainen et al. (2021), recently, have also found evidence between perceived usefulness and information adoption behaviors regarding tourism destinations. Moreover, the usefulness of information is also found to be the main reason for followership of individuals toward micro-celebrities as well as recommendation adoptions (Chen et al., 2019; Djafarova and Trofimenko, 2019). Taken together, we assume that visual aesthetics positively affects perceived usefulness, which, in turn, positively influences social commerce intentions. As such, we propose that:

H2: Perceived usefulness mediates the relationship between visual aesthetics and s-commerce intention.

#### The Moderating Role of Self-Construal

Self-construal refers to how individuals perceive, comprehend, and interpret themselves by the cognition of their relationship with the surroundings (Markus and Kitayama, 1991; Han and Humphreys, 2016). Based on the view of the self as related to or distinct from others, two types of self-construals are conceptualized, namely independent self-construal (IndSC) or InterSC (Markus and Kitayama, 1991). Existing literature suggests that self-construal is an essential personality pattern to explain the differences of individuals in perceptions, motivations, judgments, and behaviors (Markus and Kitayama, 1991; Cross et al., 2011; Haberstroh et al., 2018). Individuals with IndSC prefer to be distinct and unique and usually emphasize self-fulfillment and internal attributes. In contrast, people with InterSC are more relationship-dependent and thus focus on social connectedness, relationships, and contexts (Singelis, 1994; Cross et al., 2011). Accordingly, IndSC and InterSC individuals present significant differences in the cognitive and motivational process for decision-making and actions (Cross et al., 2011; Fang, 2017; Haberstroh et al., 2018). Specifically, IndSC individuals are more goal oriented and exert an analytic thinking style, focusing on the functional judgment of the task detached from its social context. In contrast, InterSC individuals are more socially sensitive and promote a holistic thinking style that makes judgments in consideration of social contexts (Konrath et al., 2009; Voyer and Franks, 2014; Cheek and Norem, 2017; Fang, 2017). Previous research indicated that self-construal will modulate the social information process and perceptual information processing of individuals (Liu et al., 2015). In short, individuals with different self-construals may process and respond to the same visual information distinctively (Hu et al., 2016), thus making a difference in the relationship between visual aesthetics and social commerce intention.

Recent studies have indicated that the different cognitive frameworks between the two types of self-construals may influence attention, priority, and response of individuals to visual information (Liddell et al., 2015; Liu et al., 2015). With an analytical cognitive style, IndSC individuals are oriented to focal objects and “make attribution and prediction with reference to internal properties of the objects” (Lin and Han, 2009), and thus perform better in local-level tasks, emphasizing decontextualized dimensions (Liddell et al., 2015). Alternatively, the holistic thinking InterSC individuals are more likely to attend to the contextual background and understand the visual information as an interconnected whole (Choi and Totten, 2012), and then are adept at handling global-level tasks (Liddell et al., 2015). Existing literature suggests that the nature of these perceptual processing tasks will regulate attentional control mechanisms of individuals (Hedden et al., 2008; Liddell et al., 2015; Liu et al., 2015). The bias toward local processing takes more attentional efforts for IndSC individuals to perform the global task, leading to greater brain activation in this case. Likewise, active attention occurs when InterSC individuals need to deal with local tasks (Hedden et al., 2008; Liddell et al., 2015).

As aforementioned, a visual presentation is a multifunctional object that provides aesthetic experience and valuable information in parallel (Couper et al., 2007; McCormick and Livett, 2012; Djafarova and Trofimenko, 2019). It is a “gestalt-like” visual compound that will generate optimal effect from a holistic higher-level configuration, indicating the need for global-level processing (Haberstroh et al., 2018). Hence, it can infer that automatic attention toward the visual presentation may be enough for an InterSC individual to make an evaluation (Simmonds et al., 2020). That is, InterSC individuals are more likely to prime the visual heuristics for decision-making. InterSC, therefore, should moderate the direct path from visual aesthetics to social commerce intention. Besides, visual aesthetics also delivers social information involving the “aesthetic self” and social identity of a certain micro-celebrity (Djafarova and Trofimenko, 2019). InterSC individuals represent a more flexible and variable self that would adapt their behaviors to match the social contexts (Singelis, 1994; Haberstroh et al., 2018). As a result, InterSC individuals are more likely to seek similarities and assimilate with others, thus generating more willingness to mimic and adopt a visual aesthetic suggestion (Aljukhadar et al., 2017). As such, we predict that:

H3: InterSC moderates the positive influence of visual aesthetics (VA) and social commerce intention (SCI), such that the effect is stronger when InterSC is higher.

Conversely, IndSC individuals may give active attention to the visual compound to allow the cognitive processing to analyze the internal attributes against perceptual interference (Liddell et al., 2015; Simmonds et al., 2020). As such, we predict that IndSC should moderate the indirect path through perceived usefulness. Besides, scholars suggest that IndSC individuals are goal/performance-orientated, preferring analytic judgments based on internal attributes (Cross et al., 2011; Voyer and Franks, 2014; Fang, 2017). When viewing the micro-celebrities visual aesthetic presentations, IndSC may emphasize utilitarian aspects of the information, such as style advice and the visualization of related products and services in the presentations, to support their shoppings (McCormick and Livett, 2012). With this regard, IndSC individuals may confront more perceptual conflicts between the target and interference in this kind of composite information (Liddell et al., 2015). As a result, more cognitive resources would require to deal with the visual aesthetics information that may decrease the perception of information usefulness. Moreover, IndSC individuals are self-determined, egocentric, and decontextualized. They would direct their behaviors and responses according to their inner feelings without being influenced by any surroundings (Millan and Reynolds, 2014; Fang, 2017). Accordingly, they may be less likely to take further actions by a descending perception of usefulness. Consequently, we indicate that:

H4a: IndSC weakens the positive effect of visual aesthetics on perceived usefulness (PU), such that the positive relationship between VA and PU is weaker when IndSC is higher.

H4b: IndSC negatively moderates the mediation effect of PU between VA and SCI, such that a higher IndSC leads to a weaker mediation effect of PU.

### Data Collection and Sample

We designed an online survey, including all the constructs in our proposed model and other demographic variables in the questionnaire. We focused on the Chinese market in that China is reported as one of the largest s-commerce markets in the world (Kemp, 2020). The survey was conducted through a professional survey platform (https://wj.qq.com). Then, we posted a URL of our questionnaire through “Circle of Friends” and “Group Chat” on the Wechat platform to encourage people to participate in our research as much as possible. In order to ensure the respondents are experienced with the concept of “social commerce,” we only collected data from those who had used social commerce platforms in the past 6 months.

Furthermore, we also put two simple logical questions in our survey to ensure that the respondents were conscientiously filling the survey. In exchange, we provided a ¥3 gift certificate for those who fully and responsibly completed the questionnaire. Finally, there were a total of 321 valid questionnaires we collected to be analyzed. About 70% of our respondents were females, and 58.6% of the participants were between 18 and 25 years old. Moreover, most of them (78.8%) had a bachelor's degree or above, and almost 44.2% got paid less than ¥2,000 monthly.

### Measures

All the constructs in our hypotheses were adapted from prior research with some fine-tuning to fit the social commerce context. In particular, the measures of visual aesthetics of self-presentations of micro-celebrity were modified from Park et al. (2015) and Chen et al. (2019). As perceived, usefulness was a mature construct but examined under a relatively new area; we chose to include both classic items from Davis (1989) and trendy items from Xiang et al. (2016) for our measurement. Items for social commerce intention were modified from Horng and Wu (2020). Lastly, self-construal items were adopted from Hofmann et al. (2021) and Singelis (1994). All the items were measured on a seven-point Likert Scale, ranging from 1 (strongly disagree) to 7 (strongly agree).

### Statistical Analysis

SPSS26.0, AMOS23.0, and Hayes' PROCESS for SPSS were used for data analysis. Firstly, Cronbach's alpha tested in SPSS 26.0 was used to examine the reliability of our measurement. Values of Cronbach's α were all above the standard of 0.7 (see Table 1; Hair et al., 2010). Then, a confirmatory factor analysis (CFA) was conducted in AMOS 23.0 to check the convergent and discriminant validity of the proposed model. Followed by this, the PROCESS for SPSS (Model 4) was used to test the direct effect of the visual aesthetics on social commerce intention and their indirect effect *via* perceived usefulness (Hayes, 2018). Finally, Model 8 of the PROCESS macro was used to examine the moderated mediation effect of the proposed hypotheses based on the regression bootstrapping method [5,000 bootstrap samples to estimate 95% confidence interval (CI); Hayes, 2018]. CIs excluding zero suggested the significance of indirect effects (Hayes, 2018).

**Table 1:** Main variable measurement items, reliability, and factor loadings.

**Construct Items**		**Factor loading**	**CR**	**AVE**	**α**	**Source**
**Visual aesthetics (VA)**			**0.876**	**0.703**	**0.875**	Park et al., 2015; Chen et al., 2019
VA1	The visual presentation of the micro-celebrity is visually appeal	0.850				
VA2	The way the micro-celebrity presented his/her visual contents is attractively arranged	0.878				
VA3	I am motived to watch this micro-celebrity's visual presentation for their visual aesthetics	0.784				
**Perceived usefulness (PU)**			**0.887**	**0.724**	**0.887**	Davis, 1989; Xiang et al., 2016
PU1	I find watch the visual presentations of the micro-celebrity useful for my purchase decision makings	0.854				
PU2	Watching the micro-celebrity's visual presentations can improve my shopping performance	0.828				
PU3	Watching the micro-celebrity's visual presentations make it easier for me to make purchase decisions	0.870				
**Social commerce intention (SCI)**			**0.910**	**0.772**	**0.909**	Horng and Wu, 2020
SCI1	I will consider the recommendations of the micro-celebrity when I want to shop.	0.858				
SCI2	I am willing to buy products recommended by this micro-celebrity	0.922				
SCI3	I will ask the micro-celebrity to provide me with their suggestions before I go shopping	0.854				
**Interdependent self-construal (InterSC)**			**0.836**	**0.562**	**0.836**	Singelis, 1994; Hofmann et al., 2021
InterSC1	It is important for me to maintain harmony within my group	0.712				
InterSC2	it is important to me to respect decisions made by the group	0.778				
InterSC3	I often have the feeling that my relationships with others are more important than my own accomplishments	0.798				
InterSC4	I have respect for the authority figures with whom I interact	0.706				
**Independent Self-Construal (IndSC)**			**0.849**	**0.588**	**0.845**	
IndSC1	Having a lively imagination is important to me	0.810				
IndSC2	My personal identity independent of others is very important to me	0.871				
IndSC3	I enjoy being unique and different from others in many respects	0.722				
IndSC4	Being able to take care of myself is a primary concern for me	0.644				

## Results

### Measurement Model

We used a CFA to test our measurement model composed of visual aesthetics, perceived usefulness, and social commerce intention. The results of fit indices suggest that the model has a good fit with the data (chi-square/*df* = 2.074 <3, AGFI = 0.937 >0.9, GFI = 0.966 >0.9, RMSEA = 0.058 <0.08 and the SRMR = 0.0293 <0.05, Hooper et al., 2008). The examinations of factor loadings (>0.5, Hair et al., 2010), composite reliability (CR, 0.836–0.910 >0.6), and average variance extraction (AVE, 0.562–0.772 >0.5) are presented in Table 1, which demonstrate a satisfactory level of convergent validity (Fornell and Larcker, 1981). Moreover, as suggested by Fornell and Larcker (1981), the values of AVE square root should surpass their inter-construct correlations to prove the discriminant validity of the model. The results providing evidence of high-convergent validity are presented in Table 2, along with means and SDs.

**Table 2:** Discriminant validity of the constructs, mean, and standard deviation.

	**Mean**	**SD**	**SCI**	**PU**	**VA**
Social commerce intention	4.706	1.340	**0.879**		
Perceived usefulness	4.900	1.217	0.807	**0.851**	
Visual aesthetics	4.779	1.172	0.613	0.680	**0.838**

*SD is Standard deviation, while bold numbers are the square root of the average variance extracted*.

### Direct and Indirect Effects

We used a two-step examination for the structural model. In the first step, we conducted a simple linear regression analysis in SPSS 26.0 to examine the relationship of factors with social commerce intention. The proposed model has explained 54.3% of the variance in s-commerce intention within a middle level (Hair et al., 2010). Visual aesthetics is significantly related to social commerce intention (*B* = 0.190, *p* < 0.005), thus supporting H1. Meanwhile, perceived usefulness is significantly related to social commerce intention (*B* = 0.688, *p* < 0.001). Then, a further mediation effect can be examined. In the next step, Hayes (2018) Model 4 was used to examine whether the mediation effect of perceived usefulness exists between visual aesthetics and social commerce intention. As shown in Table 3, the bootstrapping results indicate that the indirect effect of visual aesthetics (VA) on s-commerce intention (SCI) through perceived usefulness (PU) is significant (direct effect: *B* = 0.190, *p* < 0.005, 95% CI, excluding zero; indirect effect: *B* = 0.433, *p* < 0.001, 95% CI, excluding zero). Therefore, H2 is supported.

**Table 3:** Mediation results—the indirect effect of VA on SCI *via* PU (PROCESS model 4, 95% CI).

**Effect**	**B**	**SE**	**t**	**p**	**LLCI**	**ULCI**
Total effect: VA→ SCI	0.623	0.054	11.606	0.000	0.518	0.729
Direct effect: VA→ PU	0.630	0.046	13.625	0.000	0.539	0.721
PU→ SCI	0.688	0.052	13.101	0.000	0.584	0.791
VA→ SCI	0.190	0.055	3.486	0.001	0.083	0.297
**Indirect effect**	**B**	**SE**	**Z**	**P**	**BootLLCI**	**BootULCI**
VA→ PU→ SCI	0.433	0.046	9.430	0.000	0.312	0.570

*N = 321. B, Unstandardized regression coefficients; LL, Lower limit; CI, Confidence interval; UL, Upper limit; Boot, Bootstrap (sample = 5, 000)*.

### The Moderated Mediation Effect

Hayes (2018) Model 8 was applied to examine the moderating role of InterSC between VA and SCI (H3), the conditional effect of IndSC between VA and PU (H4), and the moderated mediation effect in which IndSC moderates the mediation effect of PU on the relationship between VA and SCI (H5). InterSC and IndSC were used as moderators to run Model 8 separately and finally got all the hypotheses tests regarding conditional effects. The results are all presented in Table 4.

**Table 4:** Moderated mediation analysis—self-construal (InterSC and IndSC) moderates the direct and indirect relationship between VA and SCI (PROCESS model 8, 95% CI).

	**B**	**se**	***t***	***p***	**LLCI**	**ULCI**
**Model 1: SCI as outcome**						
PU	0.607	0.057	10.575	0.000	0.494	0.720
VA	0.149	0.055	2.729	0.007	0.042	0.256
InterSC	0.235	0.059	4.017	0.000	0.120	0.350
VA X InterSC	0.055	0.026	2.102	0.036	0.004	0.107
**The conditional direct effect of VA on SCI at values of InterSC**						
InterSC (-1 SD)	0.087	0.062	1.407	0.160	−0.034	0.208
InterSC (+1 SD)	0.211	0.063	3.370	0.001	0.088	0.335
**Model 2: PU as outcome**						
VA	0.399	0.046	8.732	0.000	0.309	0.489
IndSC	0.447	0.054	8.221	0.000	0.340	0.554
VA X IndSC	−0.073	0.025	−2.884	0.004	−0.122	−0.023
**The conditional direct effect of VA on PU at values of IndSC**						
IndSC (−1 SD)	0.478	0.054	8.869	0.000	0.372	0.584
IndSC (+1 SD)	0.321	0.053	6.093	0.000	0.217	0.424
**Bootstrapping results for the indirect effect (via PU) between VA and SCI**						
**Index of moderated mediation**	**Index**	**SE (Boot)**		**BootLLCI**	**BootULCI**	
IndSC(moderator)		−0.045	0.021		−0.092	−0.009
**The conditional indirect effect of VA ON SCI via PU**						
IndSC (−1 SD)		0.295	0.062		0.185	0.427
IndSC (+1 SD)		0.198	0.067		0.091	0.355

*N = 321. B, unstandardized regression coefficients; LL, lower limit; CI, confidence interval; UL, upper limit; Boot, bootstrap (sample = 5,000); VA, visual aesthetics; PU, perceived usefulness; SCI, social commerce intention; InterSC, interdependent self-construal; IndSC, independent self-construal*.

**Figure 2:**
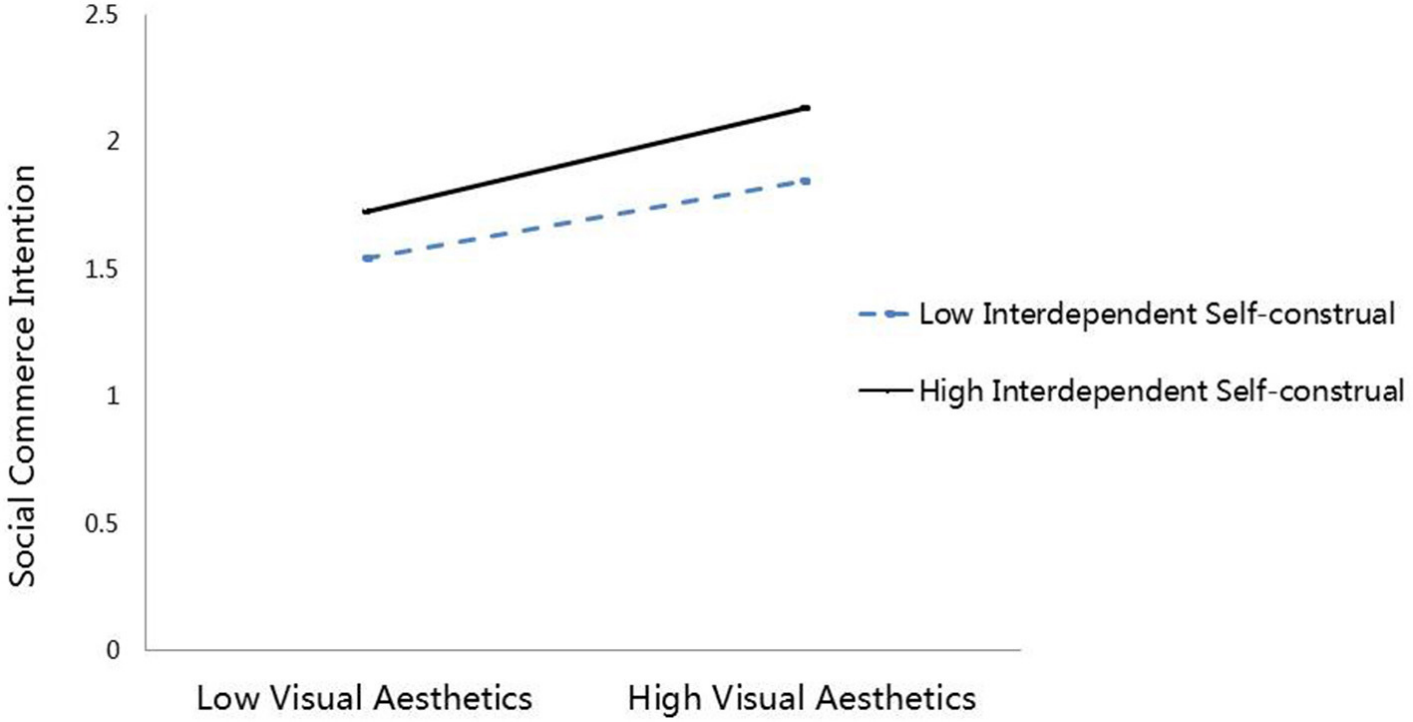
The moderating effect of interdependent self-construal between visual aesthetics and social commerce intention.

The results reveal that the interaction effect of VA × InterSCon SCI is significant (*B* = 0.055, *p* < 0.05, 95% CI, excluding zero), showing that InterSC moderates the positive relationship between VA and SCI. Aiken and West (1991) suggested that the interaction effects are plotted at ± 1 SD from the mean of InterSC (Figure 2). A simple slope test examines the strength of the relationship between VA and SCI at different (high/low) levels of InterSC. The results indicate that the conditional direct effect of VA on SCI is not significant when InterSC is low (*B* = 0.087, *p* > 0.05, 95% CI, including zero). In contrast, the conditional direct effect of VA on SCI is significant and strong (*B* = 0.211, *p* < 0.005, 95% CI, excluding zero) at a high InterSC level. H3 thus is supported.

**Figure 3:**
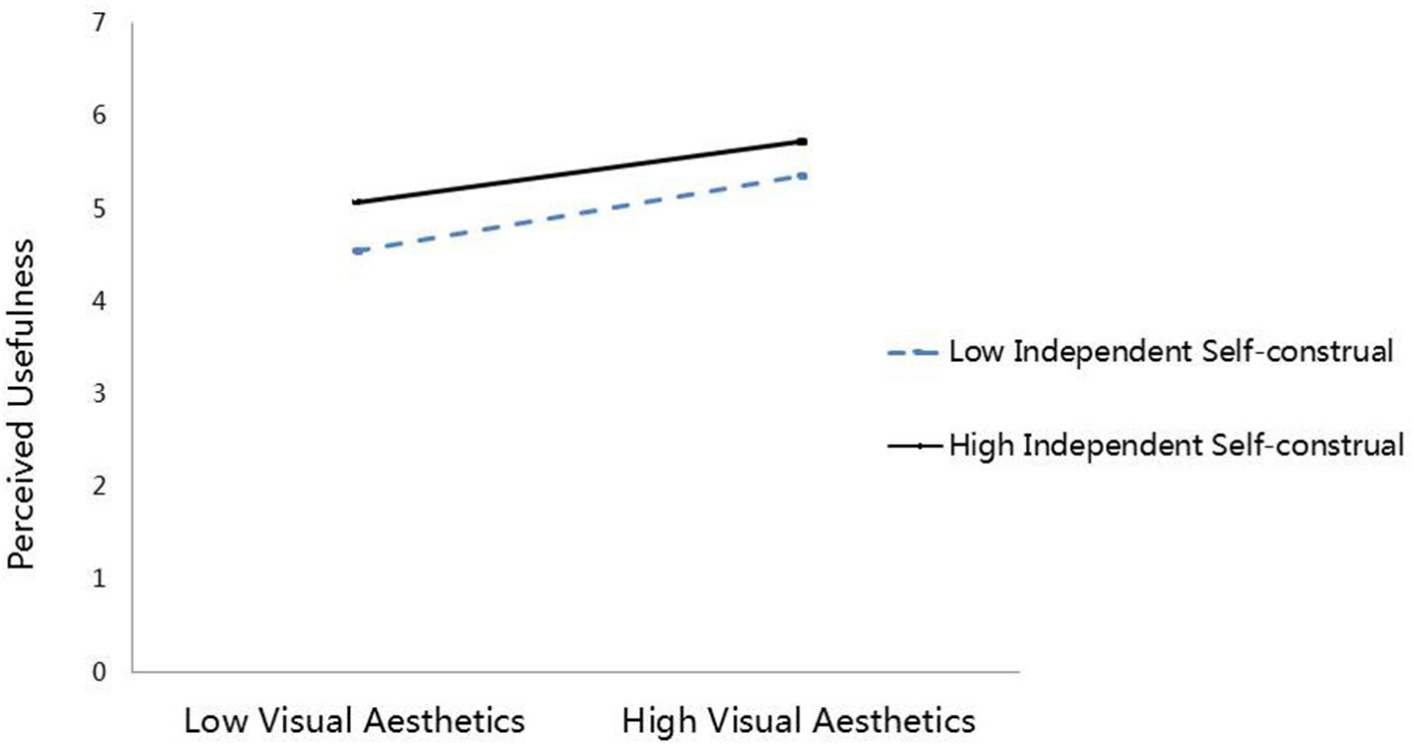
The moderating effect of independent self-construal between visual aesthetics and perceived usefulness.

The interaction effect of VA × IndSC on PU is significant (*B* = −0.073, *p* < 0.05, 95% CI, excluding zero), showing that IndSC negatively moderates the positive effect of VA on PU. Following the approach of Aiken and West (1991), these interactions are plotted at ± 1 SD from the mean of IndSC (Figure 3). The simple slope approach is used to test the strength of the relationship between VA and PU at different levels (high/low) of IndSC. The results reveal that the VA-PU relationship is strong (*B* = 0.478, *p* < 0.001, 95% CI, excluding zero) when IndSC is at a low level, while the relationship is weak (*B* = 0.321, *p* < 0.001, 95% CI, excluding zero) when IndSC is at a high level. Thus, H4a is proved. The results depict that IndSC moderates the indirect relationship between VA and SCI *via* PU (bootstrap estimate = −0.045, bias-corrected CI, excluding zero). As shown in Table 4, the conditional indirect effect (*via* PU) of VA on SCI is weaker (bootstrap estimate = 0.198, bias-corrected CI, including zero) at a high level of IndSC (+1 SD) than that at a low level of IndSC (−1 SD) (bootstrap estimate = 0.295, bias-corrected CI, including zero). Hence, H4b is supported.

## Discussion

Micro-celebrities play a significant role in the social commerce economy by encouraging the participant and purchasing behaviors of online consumers (Kádeková and Holienčinová, 2018; SanMiguel and Sádaba, 2018). The rise of visual-oriented platforms further enlarges the influence of micro-celebrities by providing them opportunities to aesthetically design and present themselves (Djafarova and Trofimenko, 2019; Jin et al., 2019), which is helpful for attention-attraction and follower-accumulation (Leaver et al., 2020; Djafarova and Bowes, 2021). In an era of “attention is profitable,” visual aesthetics is probably more important than competence or expertise for micro-celebrities popularity (Marwick, 2015). The present study, thus, provides the first attempt to empirically examine the relationship between the visual aesthetics of self-presentation and social commerce intentions of micro-celebrities with the discussions about the direct effect, intervene mechanism, and boundary conditions between them.

Our empirical findings confirm the significant role of visual aesthetic presentations in the context of social commerce. Drawn on visual heuristic theory and VIAUM, the present outcomes depict that visual aesthetics has a positive direct relationship with social commerce intention (H1) and a positive indirect influence *via* perceived usefulness (H2). We suggest that visual aesthetics can be an effective visual heuristics as a “preference” for quick and intuitive choices (Townsend and Kahn, 2014; Greussing and Boomgaarden, 2019; Ishfaq et al., 2020). Besides, it can also serve as a practical “inference” for rational judgment, which leads to better decision-making (Mahon-Haft and Dillman, 2010; Gigerenzer and Gaissmaier, 2011; Coursaris and Van Osch, 2016). The mediation mechanism of perceived usefulness found in the study clarifies how visual aesthetics information can effectively communicate to consumers and consequently influence their social commerce behaviors. Moreover, our findings reveal that self-construal makes a significant difference in the persuasion effects of visual aesthetics on social commerce intention. The results show that InterSC strengthens the positive relationship between visual aesthetics and social commerce intention (H3), while IndSC weakens this relationship by decreasing the perceived usefulness (H4a, H4b). This finding is in accord with the self-construal research in which InterSC and IndSC individuals are proved distinct in cognitive-perceptual styles. With a holistic and context-dependent cognitive style, InterSC individuals will rely more on contextual information than those IndSC people with an analytic and decontextualized cognitive style when making decisions (Lin et al., 2008; Cross et al., 2011; Liu et al., 2015). As InterSC individuals are biased in local processing, visual aesthetic presentations containing an implication of aesthetic advice or product-related visualization will exert active attention of IndSC individuals to diagnose the visual cues for rational decisions (Liddell et al., 2015; Simmonds et al., 2020). Consequently, as more attentional resources and cognitive efforts are required to process it, the perceived usefulness of visual aesthetics decreases, and then social commerce intention follows for IndSC individuals (Liddell et al., 2015; Fang, 2017).

In short, this study suggests that visual aesthetics is an effective self-presentation strategy that will encourage the social commerce intention of online consumers. Previous studies announced that the visual aesthetics of salespeople or micro-celebrities cannot directly influence the purchase decisions of consumers (Ahearne et al., 1999; Djafarova and Trofimenko, 2019). Conversely, based on heuristic theory, our empirical research verifies that visual aesthetics serves as a low-resources shortcut that leads to direct decision-making. Besides, online consumers nowadays are fed up with the commercial-driven “advertorials” on social media. They perceive these ads as compelling persuasion knowledge manipulated by the brands or presenters and, therefore, would not take them as the source of information (Singh et al., 2020). In the lens of visual communication, we suggest that the visual aesthetics of self-presentation of micro-celebrity is a compound visual information that can attract attention, provide aesthetic experiences, and convey useful information. This multifunctional information is more ambiguous in promotional intentions and less compulsive (in persuasion) and thus generates a better persuasion effect than the commercial-related posts (Rietveld et al., 2020). It is worth mentioning that, although the power of visual aesthetics increases in the InterSC group, its perceived usefulness diminishes in the IndSC group and thus affects the ultimate social commerce intention. Micro-celebrities need to consider the comprehensive characteristics of the target audiences when conducting a visual aesthetics strategy. That is, they need to maintain and enhance this visual aesthetics within the followers with InterSC. Nevertheless, they should work on increasing the perceived usefulness of the IndSC followers by offering more attribute-related cues.

### Theoretical Contributions

The present research contributes to the literature in at least three aspects. First, previous studies on visual aesthetics have been limited to the web-system perspectives due to the emphasis of e-commerce on maximizing efficiency (Lorenzo-Romero et al., 2013; Chen et al., 2018; Longstreet et al., 2021). We extend this study to the social commerce domain by emphasizing the visual aesthetics effects from the perspectives of visual presentations of micro-celebrities. Besides, by viewing visual aesthetic presentations as means of visual communication, intervening aesthetic experience with knowledge dissemination, we expand the scope of the visual aesthetics of micro-celebrities from physical attractiveness to strategic communication (Couper et al., 2007; Berube, 2019; Seifert and Chattaraman, 2020).

In addition, we introduce a VIAUM for the first time to explore the underlying mechanism through which visual aesthetics affects social commerce intention. By highlighting the prominent role of visual heuristics, VIAUM provides a practical and flexible framework to understand the persuasive process of contextual information in the digital world (Gigerenzer and Gaissmaier, 2011; Saab and Botelho, 2020). Previous studies generally held a principle that central arguments usually override the judgmental influences of visual heuristics. Besides, visual aesthetics is likely to shape cognitive bias and thus drives irrational decisions (Ishfaq et al., 2020). Similarly, visual aesthetics is usually considered as mood-related cues that influence responses of consumers based on an affective system (Kim and Lennon, 2008; Phelan et al., 2011). Our VIAUM implies that visual aesthetics (as heuristics) is functionally equivalent with central arguments that provide essential evidence for cognitive inferences. Similar to the dual-code audiovisual cues, visual aesthetic presentations can elicit the automatic attention of individuals as an external stimulus and generate their internal processing as a message of implicit meaning (Simmonds et al., 2020). By confirming a reasoning mechanism (*via* perceived usefulness) between visual aesthetics and behavioral responses of consumers, our empirical results of VIAUM thus explain how visual information makes sense to consumers. In short, the developed and empirically validated VIAUM leads to a shift in the understanding of visual communication in the social commerce environment from the descriptive level to conceptual and explanatory levels (Wang and Zhang, 2012).

Last but not least, we innovatively incorporated self-construal to explain the differences of the individuals in visual persuasion effects. Our findings indicate that self-construct orientation of people, namely InterSC and IndSC, significantly affect direct and indirect visual aesthetics (*via* perceived usefulness) effects on social commerce intention of individuals. By focusing the persuasive process of visual information on motivation and cognitive ability rather than information types, the VIAUM framework conforms to the principle of “triadic reciprocal determinism” in which psychological cognition is regarded as a complicated process of the “triangle interaction” of contexts, individuals, and behaviors (Bandura, 1986). As such, VIAUM allows an in-depth but straightforward exploration of visual communication strategy in the context of social commerce. VIAUM, therefore, responds to the call of McNamara and Houston (McNamara and Houston, 2009, p. 673) of building “simple mechanisms that will evolve in complex environments” rather than “complex models of optimal behavior in simple environments.”

### Managerial Implications

Beyond our theoretical development, the present study may serve as guidance for social-commerce operators, brand managers, and micro-celebrities. First, we suggest that visual aesthetic strategy is a prerequisite for visual communication in the context of social commerce. According to our results, visual aesthetics can promote the direct social commerce intentions of consumers. Meanwhile, visual aesthetics facilitates the perceived usefulness of the visual information, encouraging consumers to take further actions regarding social commerce. In short, for business conducted in such a visually dominated environment, it is necessary to apply aesthetic skills in their visual presentations to attract attention and influence consumers for ultimate commercial ends. Besides, based on VIAUM and related theories, we suggest that visual aesthetics means more than physical attractiveness. It implies that brand managers should cooperate with micro-celebrities who can aesthetically present their contents rather than merely good-looking ones.

However, although visual aesthetics is effective in social commerce marketing, its influence varies significantly from person to person. According to our findings, the effect of visual aesthetics is more evident for people with InterSC regarding their direct social commerce intentions. Moreover, for people with independent self-construal, the role of visual aesthetic strategies in the usefulness perception and the further responses is even weakened. Therefore, we suggest that personalized visual aesthetic strategies should be implemented for consumers with different consumption demands. Specifically, for those consumers with InterSC traits, sustainable and enhanced visual aesthetics strategies are encouraged. However, for those consumers with IndSC, the visual aesthetics strategy should be carefully conducted or collocate with more attribute implications.

### Limitations and Future Research Directions

This study is not without its limitations. First, most of the respondents to this research were females (70%) and university students (58.6%), limiting the generality of the results. According to Wang and Zhang (2012), women are more interested in social networks than men, making social commerce a female-dominated business. In this respect, our findings may be quite different in the male population. Besides, Kádeková and Holienčinová (2018) indicate that the social networks and visual contents are more prevalent in millennials. Alternative effects may be generated from people of different age stages. Therefore, future research should be conducted in a larger scope of social groups to further verify the linkages between visual aesthetic communication and social commerce intentions.

Additionally, selection bias may influence our results in that the present study does not define product types. Past studies indicated that evaluations, attitudes, and decision-making of consumers vary between hedonic products and utilitarian products (Dhar and Wertenbroch, 2000; Lee et al., 2012). Especially, hedonic goods are perceived to be more meaning carrying and social expressive, while utilitarian goods are more instrumental and functional (Dhar and Wertenbroch, 2000; Lee et al., 2012). As a result, the positive interaction effect of visual aesthetics and InterSC on social commerce intention may be further strengthened by presentations with hedonic products than utilization products. On the contrary, the negative interaction effect of visual aesthetic and IndSC on perceived usefulness may be mitigated in the context of utilization-product-related presentations. At this point, there is a need for further examination.

Furthermore, the measurement of visual aesthetics in the present research is based on the overall aesthetic impression of visual presentations. Visual aesthetics may result from different visual rhetoric techniques (Schroeder, 2004; Yang et al., 2010; Leaver et al., 2020). Previous studies claim that presentation styles significantly influence product perceptions of consumers and the effectiveness of an advertisement (Yang et al., 2010). Visual presentations with multiple modalities, such as texts with pictures (Toepoel and Couper, 2011) and audio with visual images (Kusumasondjaja, 2019; Simmonds et al., 2020), will facilitate understanding and perceptions of consumers of the visual information and thus generate more positive responses than simple and plain modalities (Toepoel and Couper, 2011; Kusumasondjaja, 2019). However, Townsend and Kahn (2014) indicate that a content-rich visual presentation may cause a problem of choice overload and thus generate negative consumer responses. Especially, DeRosia and McQuarrie (2019) find that different propensity of individuals to process visual information (VisProp) will elicit different evaluations of the visual aesthetics toward a visual advertisement. For these reasons, further exploration is needed for the antecedents of visual aesthetics perceptions.

## Conclusion

By conducting a VIAUM to examine the relationships between visual aesthetics of self-presentation of micro-celebrities and intention of consumers for social commerce, the main conclusions of the present research should be three-fold. First, we suggest that visual aesthetics will directly influence social commerce intentions of individuals. Second, an intervening mechanism confirms that visual aesthetics will indirectly affect social commerce intentions through perceived usefulness. Finally, as human behavior intentions may form from external and internal causes, this study also explores the interaction effects of visual aesthetics and self-construal of individuals on social commerce intentions. According to our findings, the positive effect of visual aesthetics on social commerce intention is more substantial for InterSC individuals. Meanwhile, people with a higher level of IndSC lead to a decreasing effect of visual aesthetics on perceived usefulness and further weaken the indirect effect between visual aesthetics and social commerce intention.

## Data Availability Statement

The raw data supporting the conclusions of this article will be made available by the authors, without undue reservation.

## Author Contributions

All the authors listed have made a substantial, direct and intellectual contribution to the work, and approved it for publication.

## Conflict of Interest

The authors declare that the research was conducted in the absence of any commercial or financial relationships that could be construed as a potential conflict of interest.

## Publisher's Note

All claims expressed in this article are solely those of the authors and do not necessarily represent those of their affiliated organizations, or those of the publisher, the editors and the reviewers. Any product that may be evaluated in this article, or claim that may be made by its manufacturer, is not guaranteed or endorsed by the publisher.

## References

[B1] Abidin C. (2016). Visibility labour: engaging with Influencers'fashion brands and #OOTD advertorial campaigns on Instagram. Media Int. Aust. 161, 86–100. 10.1177/1329878X16665177

[B2] Ahearne M. Gruen T. W. Jarvis C. B. (1999). If looks could sell: moderation and mediation of the attractiveness effect on salesperson performance. Int. J. Res. Market. 16, 269–284. 10.1016/S0167-8116(99)00014-2

[B3] Ahluwalia R. (2008). How far can a brand stretch? Understanding the role of self-construal. J. Market. Res. 45, 337–350. 10.1509/jmkr.45.3.337

[B4] Aiken L. S. West S. G. (1991). Multiple Regression: Testing and Interpreting Interactions. Thousand Oaks, CA: Sage Publications, Inc.

[B5] Al-Emadi F. A. Ben Yahia I. (2020). Ordinary celebrities related criteria to harvest fame and influence on social media. J. Res. Interact. Market. 14, 195–213. 10.1108/JRIM-02-2018-0031

[B6] Aljukhadar M. Bériault Poirier A. Senecal S. (2020). Imagery makes social media captivating! Aesthetic value in a consumer-as-value-maximizer framework. J. Res. Interact. Market. 14, 285–303. 10.1108/JRIM-10-2018-0136

[B7] Aljukhadar M. Trifts V. Senecal S. (2017). Consumer self-construal and trust as determinants of the reactance to a recommender advice. Psychol. Market. 34, 708–719. 10.1002/mar.21017

[B8] Bandura A. (1986). The explanatory and predictive scope of self-efficacy theory. J. Soc. Clin. Psychol. 4, 359–373. 10.1521/jscp.1986.4.3.359

[B9] Berube D. (2019). Visual communication and heuristics: challenges and directions from across the disciplines. OSF Preprints. 2019, 2–3. 10.31219/osf.io/bqjpf

[B10] Bhandari U. Chang K. Neben T. (2019). Understanding the impact of perceived visual aesthetics on user evaluations: an emotional perspective. Inform. Manage. 56, 85–93. 10.1016/j.im.2018.07.003

[B11] Braun J. Amirshahi S. A. Denzler J. Redies C. (2013). Statistical image properties of print advertisements, visual artworks and images of architecture. Front. Psychol. 4:808. 10.3389/fpsyg.2013.00808 24204353 PMC3817471

[B12] Brydges T. Sjöholm J. (2019). Becoming a personal style blogger: changing configurations and spatialities of aesthetic labour in the fashion industry. Int. J. Cult. Stud. 22, 119–139. 10.1177/1367877917752404

[B13] Busalim A. H. Hussin A. R. C. (2016). Understanding social commerce: a systematic literature review and directions for further research. Int. J. Inf. Manage. 36, 1075–1088. 10.1016/j.ijinfomgt.2016.06.005

[B14] Cheek N. N. Norem J. K. (2017). Holistic thinkers anchor less: exploring the roles of self-construal and thinking styles in anchoring susceptibility. Pers. Individ. Dif. 115, 174–176. 10.1016/j.paid.2016.01.034

[B15] Chen C. C. Hsiao K. L. Wu S. J. (2018). Purchase intention in social commerce: an empirical examination of perceived value and social awareness. Libr. Hi Tech. 36, 583–604. 10.1108/LHT-01-2018-0007

[B16] Chen J. Shen X. L. (2015). Consumers'decisions in social commerce context: an empirical investigation. Decis. Support Syst. 79, 55–64. 10.1016/j.dss.2015.07.012

[B17] Chen Y. Lu Y. Wang B. Pan Z. (2019). How do product recommendations affect impulse buying? An empirical study on WeChat social commerce. Inform. Manage. 56, 236–248. 10.1016/j.im.2018.09.002

[B18] Choi Y. K. Totten J. W. (2012). Self-construal's role in mobile TV acceptance: extension of TAM across cultures. J. Bus. Res. 65, 1525–1533. 10.1016/j.jbusres.2011.02.036

[B19] Chu S.-C. Kamal S. (2008). The effect of perceived blogger credibility and argument quality on message elaboration and brand attitudes. J. Interact. Advert. 8, 26–37. 10.1080/15252019.2008.10722140

[B20] Chung N. Han H. Koo C. (2015). Adoption of travel information in user-generated content on social media: the moderating effect of social presence. Behav. Inform. Technol. 34, 902–919. 10.1080/0144929X.2015.1039060

[B21] Couper M. P. Conrad F. G. Tourangeau R. (2007). Visual context effects in web surveys. Public Opin. Q. 71, 623–634. 10.1093/poq/nfm044 PMC3954164 24634546

[B22] Coursaris C. K. Van Osch W. (2016). A Cognitive-Affective Model of Perceived User Satisfaction (CAMPUS): the complementary effects and interdependence of usability and aesthetics in IS design. Inform. Manage. 53, 252–264. 10.1016/j.im.2015.10.003

[B23] Cross S. E. Hardin E. E. Gercek-Swing B. (2011). The what, how, why, and where of self-construal. Pers. Soc. Psychol. Rev. 15, 142–179. 10.1177/1088868310373752 20716643

[B24] Davis F. D. (1989). Perceived usefulness, perceived ease of use, and user acceptance of information technology. MIS Quar. 13, 319–340. 10.2307/249008

[B25] DeRosia E. D. McQuarrie E. F. (2019). Lost and found: individual differences in propensity to process visual elements of persuasion. Psychol. Market. 36, 266–275. 10.1002/mar.21177

[B26] Dhar R. Wertenbroch K. (2000). Consumer choice between hedonic and utilitarian goods. J. Market. Res. 37, 60–71. 10.1509/jmkr.37.1.60.18718

[B27] Djafarova E. Bowes T. (2021). ‘Instagram made Me buy it’: generation Z impulse purchases in fashion industry. J. Retail. Consum. Serv. 59:102345. 10.1016/j.jretconser.2020.102345

[B28] Djafarova E. Trofimenko O. (2019). ‘Instafamous’–credibility and self-presentation of micro-celebrities on social media. Inform. Commun. Soc. 22, 1432–1446. 10.1080/1369118X.2018.1438491

[B29] Erkan I. Evans C. (2016). The influence of eWOM in social media on consumers'purchase intentions: an extended approach to information adoption. Comput. Human Behav. 61, 47–55. 10.1016/j.chb.2016.03.003

[B30] Fang Y.-H. (2017). Beyond the usefulness of branded applications: insights from consumer-brand engagement and self-construal perspectives. Psychol. Market. 34, 40–58. 10.1002/mar.20972

[B31] Fornell C. Larcker D. F. (1981). Evaluating structural equation models with unobservable variables and measurement error. J. Market. Res. 18, 39–50. 10.1177/002224378101800104

[B32] Fu S. Xu Y. Yan Q. (2019). Enhancing the parasocial interaction relationship between consumers through similarity effects in the context of social commerce: evidence from social commerce platforms in China. J. Strat. Market. 27, 100–118. 10.1080/0965254X.2017.1384045

[B33] García-Rapp F. (2017). Popularity markers on YouTube's attention economy: the case of Bubzbeauty. Celebr. Stud. 8, 228–245. 10.1080/19392397.2016.1242430

[B34] Gigerenzer G. Gaissmaier W. (2011). Heuristic decision making. Annu. Rev. Psychol. 62, 451–482. 10.1146/annurev-psych-120709-145346 21126183

[B35] Giles D. C. Edwards L. (2018). Instagram and the Rise of the social media ‘Influencer’, in Twenty-First Century Celebrity: Fame In Digital Culture, ed Giles D. C. (Bingley: Emerald Publishing Limited), 155–173. 10.1108/978-1-78743-708-120181012

[B36] Greussing E. Boomgaarden H. G. (2019). Simply bells and whistles?: cognitive effects of visual aesthetics in digital longforms. Digit. J. 7, 273–293. 10.1080/21670811.2018.1488598

[B37] Haberstroh K. Orth U. R. Bouzdine-Chameeva T. Cohen J. Maria Corsi A. Crouch R.. (2018). Through the lens of self-construal: cross-cultural variation in consumers'appreciation of harmony in marketing visuals. Int. Market. Rev. 35, 429–457. 10.1108/IMR-12-2015-0283

[B38] Hair J. R. J. F. Black W. C. Babin B. J. Anderson R. E. (2010). Multivaritate Data Analysis, 7th Edn. Hoboken, NJ: Pearson Prentice Hall.

[B39] Hajli M. (2013). A research framework for social commerce adoption. Inform. Manage. Comput. Secur. 21, 144–154. 10.1108/IMCS-04-2012-0024

[B40] Hajli N. Sims J. (2015). Social commerce: the transfer of power from sellers to buyers. Technol. Forecast. Soc. Change 94, 350–358. 10.1016/j.techfore.2015.01.012

[B41] Han H. Xu H. Chen H. (2018). Social commerce: a systematic review and data synthesis. Electron. Commer. Res. Appl. 30, 38–50. 10.1016/j.elerap.2018.05.005

[B42] Han S. Humphreys G. (2016). Self-construal: a cultural framework for brain function. Curr. Opin. Psychol. 8, 10–14. 10.1016/j.copsyc.2015.09.013 29506783

[B43] Harris B. R. (2006). Visual information literacy via visual means: three heuristics. Refer. Serv. Rev. 34, 213–221. 10.1108/00907320610669452

[B44] Harris L. C. Goode M. M. H. (2010). Online servicescapes, trust, and purchase intentions. J. Serv. Market. 24, 230–243. 10.1108/08876041011040631

[B45] Hayes A. F. (2018). Introduction to Mediation, Moderation, and Conditional Process Analysis-A Regression-Based Approach, 2nd. Edn. New York: The Guilford Press.

[B46] Hedden T. Ketay S. Aron A. Markus H. R. Gabrieli J. D. E. (2008). Cultural influences on neural substrates of attentional control. Psychol. Sci. 19, 12–17. 10.1111/j.1467-9280.2008.02038.x 18181784

[B47] Hofmann V. Schwayer L. M. Stokburger-Sauer N. E. Wanisch A. T. (2021). Consumers'self-construal: measurement and relevance for social media communication success. J. Consum. Behav. 2020, 1–21. 10.1002/cb.1927

[B48] Hooper D. Coughlan J. Mullen M. R. (2008). Structural equation modelling: guidelines for determining model fit. Electr. J. Bus. Res. Methods 6, 53–60. 10.0000/PMID35188134

[B49] Horn R. E. (1998). Visual Language: Global Communication for the 21 st Century. Bainbridge Island, WA: Macro VU, Inc.

[B50] Horng S. M. Wu C. L. (2020). How behaviors on social network sites and online social capital influence social commerce intentions. Inform. Manage. 57:103176. 10.1016/j.im.2019.103176

[B51] Hu M. Zhang M. Luo N. (2016). Understanding participation on video sharing communities: the role of self-construal and community interactivity. Comput. Human Behav. 62, 105–115. 10.1016/j.chb.2016.03.077

[B52] Ishfaq M. Nazir M. S. Qamar M. A. J. Usman M. (2020). Cognitive bias and the extraversion personality shaping the behavior of investors. Front. Psychol. 11:556506. 10.3389/fpsyg.2020.556506 33178066 PMC7593711

[B53] Jin S. V. Muqaddam A. Ryu E. (2019). Instafamous and social media influencer marketing. Market. Intell. Plann. 37, 567–579. 10.1108/MIP-09-2018-0375

[B54] Jin S. V. Ryu E. (2019). Instagram fashionistas, luxury visual image strategies and vanity. J. Prod. Brand Manage. 29, 355–368. 10.1108/JPBM-08-2018-1987

[B55] Kádeková Z. Holienčinová M. (2018). Influencer marketing as a modern phenomenon creating a new frontier of virtual opportunities. Commun. Today 9, 90–104. Retrieved from: https://www.researchgate.net/publication/329247338_Influencer_marketing_as_a_modern_phenomenon_creating_a_new_frontier_of_virtual_opportunities

[B56] Kemp S. (2020). Digital 2020: Global Digital Overview. Vancouver, BC: Hootsuite.

[B57] Ki C. W. C. Cuevas L. M. Chong S. M. Lim H. (2020). Influencer marketing: social media influencers as human brands attaching to followers and yielding positive marketing results by fulfilling needs. J. Retail. Consum. Serv. 55:102133. 10.1016/j.jretconser.2020.102133

[B58] Ki C. W. C. Kim Y. K. (2019). The mechanism by which social media influencers persuade consumers: the role of consumers'desire to mimic. Psychol. Market. 36, 905–922. 10.1002/mar.21244

[B59] Kim M. Lennon S. (2008). The effects of visual and verbal information on attitudes and purchase intentions in internet shopping. Psychol. Market. 25, 146–178. 10.1002/mar.20204

[B60] Konrath S. Bushman B. J. Grove T. (2009). Seeing my world in a million little pieces: narcissism, self-construal, and cognitive-perceptual style. J. Pers. 77, 1197–1228. 10.1111/j.1467-6494.2009.00579.x 19558438

[B61] Kruglanski A. W. Thompson E. P. (1999). Persuasion by a single route: a view from the unimodel. Psychol. Inq. 10, 83–109. 10.1207/S15327965PL100201

[B62] Kusumasondjaja S. (2019). Exploring the role of visual aesthetics and presentation modality in luxury fashion brand communication on Instagram. J. Fash. Market. Manage. 24, 15–31. 10.1108/JFMM-02-2019-0019

[B63] Leaver T. Highfield T. Abidin C. (2020). Instagram:Visual Social Media Cultures. Cambridge, UK; Medford, MA Polity Press.

[B64] Lee D. Kim H. S. Kim J. K. (2012). The role of self-construal in consumers'electronic word of mouth (eWOM) in social networking sites: a social cognitive approach. Comput. Human Behav. 28, 1054–1062. 10.1016/j.chb.2012.01.009

[B65] Lee J. E. Watkins B. (2016). YouTube vloggers' influence on consumer luxury brand perceptions and intentions. J. Bus. Res. 69, 5753–5760. 10.1016/j.jbusres.2016.04.171

[B66] Liang T. P. Turban E. (2011). Introduction to the special issue social commerce: a research framework for social commerce. Int. J. Electr. Commer. 16, 5–13. 10.2753/JEC1086-4415160201

[B67] Liddell B. J. Das P. Battaglini E. Malhi G. S. Felmingham K. L. Whitford T. J.. (2015). Self-orientation modulates the neural correlates of global and local processing. PLoS ONE 10:135453. 10.1371/journal.pone.0135453 26270820 PMC4536227

[B68] Lin Z. Han S. (2009). Self-construal priming modulates the scope of visual attention. Quar. J. Exp. Psychol. 62, 802–813. 10.1080/17470210802271650 18720280

[B69] Lin Z. Lin Y. Han S. (2008). Self-construal priming modulates visual activity underlying global/local perception. Biol. Psychol. 77, 93–97. 10.1016/j.biopsycho.2007.08.002 17884279

[B70] Liu Y. Li H. Hu F. (2013). Website attributes in urging online impulse purchase: an empirical investigation on consumer perceptions. Decis. Support Syst. 55, 829–837. 10.1016/j.dss.2013.04.001

[B71] Liu Z. Cheng M. Peng K. Zhang D. (2015). Self-construal priming selectively modulates the scope of visual attention. Front. Psychol. 6:1508. 10.3389/fpsyg.2015.01508 26483747 PMC4588108

[B72] Longstreet P. Valacich J. Wells J. (2021). Towards an understanding of online visual aesthetics: an instantiation of the composition perspective. Technol. Soc. 65:101536. 10.1016/j.techsoc.2021.101536

[B73] Lorenzo-Romero C. Constantinides E. Alarcón-del-Amo M. del C. (2013). Web aesthetics effects on user decisions: impact of exposure length on website quality perceptions and buying intentions. J. Internet Commer. 12, 76–105. 10.1080/15332861.2013.763695

[B74] Lucker G. W. Beane W. E. Helmreich R. L. (1981). The strength of the halo effect in physical attractiveness research. J. Psychol. Interdiscipl. Appl. 107, 69–75. 10.1080/00223980.1981.9915206

[B75] Mahon-Haft T. A. Dillman D. A. (2010). Does visual appeal matter? Effects of web survey aesthetics on survey quality. Surv. Res. Methods 4, 45–59. 10.18148/srm/2010.v4i1.2264

[B76] Markus H. R. Kitayama S. (1991). Culture and the self: implications for cognition, emotion, and motivation. Psychol. Rev. 98, 224–253. 10.1037/0033-295X.98.2.224

[B77] Marwick A. E. (2015). Instafame: luxury selfies in the attention economy. Publ. Cult. 27, 137–160. 10.1215/08992363-2798379

[B78] McCormick H. Livett C. (2012). Analysing the influence of the presentation of fashion garments on young consumers'online behaviour. J. Fash. Market. Manage. 16, 21–41. 10.1108/13612021211203014

[B79] McFarlane A. Samsioe E. (2020). #50+ fashion Instagram influencers: cognitive age and aesthetic digital labours. J. Fash. Market. Manage. 24, 399–413. 10.1108/JFMM-08-2019-0177

[B80] McNamara J. M. Houston A. I. (2009). Integrating function and mechanism. Trends Ecol. Evol. 24, 670–675. 10.1016/j.tree.2009.05.011 19683827

[B81] McQuarrie E. F. Miller J. Phillips B. J. (2013). The megaphone effect: taste and audience in fashion blogging. J. Consum. Res. 40, 136–158. 10.1086/669042

[B82] Millan E. Reynolds J. (2014). Self-construals, symbolic and hedonic preferences, and actual purchase behavior. J. Retail. Consum. Serv. 21, 550–560. 10.1016/j.jretconser.2014.03.012

[B83] Mudambi S. M. Schuff D. (2010). Research note: What makes a helpful online review? a study of customer reviews on Amazon.com. MIS Q. 34, 185–200. 10.2307/20721420

[B84] Pallak S. R. Murroni E. Koch J. (1983). Communicator attractiveness and expertise, emotional versus rational appeals, and persuasion: a heuristic versus systematic processing interpretation. Soc. Cogn. 2, 122–141. 10.1521/soco.1983.2.2.122

[B85] Palmer C. L. Peterson R. D. (2016). Halo effects and the attractiveness premium in perceptions of political expertise. Amer. Polit. Res. 44, 353–382. 10.1177/1532673X15600517

[B86] Park H. H. Jeon J. O. Sullivan P. (2015). How does visual merchandising in fashion retail stores affect consumers'brand attitude and purchase intention? Int. Rev. Retail Distrib. Consum. Res. 25, 87–104. 10.1080/09593969.2014.918048

[B87] Pedroni M. (2017). Meso-celebrities, fashion and the media: how digital influencers struggle for visibility. Film Fash. Consump. 5, 103–121. 10.1386/ffc.5.1.103_1

[B88] Phelan K. V. Christodoulidou N. Countryman C. C. Kistner L. J. (2011). To book or not to book: the role of hotel web site heuristics. J. Serv. Market. 25, 134–148. 10.1108/08876041111119859

[B89] Rietveld R. van Dolen W. Mazloom M. Worring M. (2020). What you feel, is what you like influence of message appeals on customer engagement on instagram. J. Interact. Market. 49, 20–53. 10.1016/j.intmar.2019.06.003

[B90] Saab A. B. Botelho D. (2020). Are organizational buyers rational? Using price heuristics in functional risk judgment. Indus. Market. Manage. 85, 141–151. 10.1016/j.indmarman.2019.10.001

[B91] Sanchez-Franco M. J. Rondan-Cataluña F. J. (2010). Virtual travel communities and customer loyalty: customer purchase involvement and web site design. Electron. Commer. Res. Appl. 9, 171–182. 10.1016/j.elerap.2009.05.004

[B92] SanMiguel P. Sádaba T. (2018). Nice to be a fashion blogger, hard to be influential: an analysis based on personal characteristics, knowledge criteria, and social factors. J. Glob. Fash. Market. 9, 40–58. 10.1080/20932685.2017.1399082

[B93] Schnurr B. Brunner-Sperdin A. Stokburger-Sauer N. E. (2017). The effect of context attractiveness on product attractiveness and product quality: the moderating role of product familiarity. Mark. Lett. 28, 241–253. 10.1007/s11002-016-9404-3

[B94] Schroeder J. (2013). Snapshot aesthetics and the strategic imagination. Invisible Cult. 18. Retrieved from: http://ivc.lib.rochester.edu/snapshot-aesthetics-and-the-strategic-imagination/

[B95] Schroeder J. E. (2004). Visual consumption in the image economy, in Elusive Consumption, 1st Edn., eds Ekstrom K. M. Brembeck H. (Oxford: Berg), 229–244.

[B96] Schroeder J. E. (2011). Style and strategy: snapshot aesthetic in brand culture, in Imagining Organizations: Performative Imagery in Business and Beyond, 1st Edn., eds Quattrone P. Thrigt N. Mclean C. Puyou F.-R. (New York, NY: Routledge). 10.4324/9780203807903

[B97] Seifert C. Chattaraman V. (2020). A picture is worth a thousand words! How visual storytelling transforms the aesthetic experience of novel designs. J. Prod. Brand Manage. 29, 913–926. 10.1108/JPBM-01-2019-2194

[B98] Simmonds L. Bogomolova S. Kennedy R. Nenycz-Thiel M. Bellman S. (2020). A dual-process model of how incorporating audio-visual sensory cues in video advertising promotes active attention. Psychol. Market. 37, 1057–1067. 10.1002/mar.21357

[B99] Simon H. A. (1955). A behavioral model of rational choice. Q. J. Econ. 69, 99–118. 10.2307/1884852

[B100] Singelis T. M. (1994). The measurement of independent and interdependent self-construals. Pers. Soc. Psychol. Bull. 20, 580–591. 10.1177/0146167294205014

[B101] Singh J. Crisafulli B. Quamina L. T. Xue M. T. (2020). 'To trust or not to trust': the impact of social media influencers on the reputation of corporate brands in crisis. J. Bus. Res. 2020, 464–480. 10.1016/j.jbusres.2020.03.039

[B102] Sokolova K. Kefi H. (2020). Instagram and YouTube bloggers promote it, why should I buy? How credibility and parasocial interaction influence purchase intentions. J. Retail. Consum. Serv. 53:101742. 10.1016/j.jretconser.2019.01.011

[B103] Stockemer D. Praino R. (2017). Physical attractiveness, voter heuristics and electoral systems: the role of candidate attractiveness under different institutional designs. Bri. J. Polit. Int. Relat. 19, 336–352. 10.1177/1369148116687533

[B104] Sussman S. W. Siegal W. S. (2003). Informational influence in organizations: an integrated approach to knowledge adoption. Inform. Syst. Res. 14, 47–65. 10.1287/isre.14.1.47.14767

[B105] Tapanainen T. Dao T. K. Nguyen T. T. H. (2021). Impacts of online word-of-mouth and personalities on intention to choose a destination. Comput. Hum. Behav. 116:106656. 10.1016/j.chb.2020.106656

[B106] Toepoel V. Couper M. P. (2011). Can verbal instructions counteract visual context effects in web surveys? Public Opin. Q. 75, 1–18. 10.1093/poq/nfq044

[B107] Toepoel V. Dillman D. A. (2011). Words, numbers, and visual heuristics in web surveys: is there a hierarchy of importance? Soc. Sci. Comput. Rev. 29, 193–207. 10.1177/0894439310370070

[B108] Tonder G. Van Spehar B. (2013). The aesthetic appeal of visual qualities, in Handbook of Experimental Phenomenology: Visual Perception of Shape, Space and Appearance, ed Albertazzi L. (Hoboken, NJ: Wiley-Blackwell), 395–414. 10.1002/9781118329016.ch16

[B109] Townsend C. Kahn B. E. (2014). The “visual preference heuristic”: the influence of visual versus verbal depiction on assortment processing, perceived variety, and choice overload. J. Consum. Res. 40, 993–1015. 10.1086/673521

[B110] Tractinsky N. Katz A. S. Ikar D. (2000). What is beautiful is usable. Interact. Comput. 13, 127–145. 10.1016/S0953-5438(00)00031-X

[B111] Tsai S. P. (2007). Message framing strategy for brand communication. J. Advert. Res. 47, 364–377. 10.2501/S0021849907070377

[B112] Voyer B. G. Franks B. (2014). Toward a better understanding of self-construal theory: an agency view of the processes of self-construal. Rev. Gen. Psychol. 18, 101–114. 10.1037/gpr0000003

[B113] Wang C. Zhang P. (2012). The evolution of social commerce : the people, management, technology, and information dimensions. Commun. Assoc. Inform. Syst. 31, 105–127. 10.17705/1CAIS.03105

[B114] Wang W. Chen R. R. Ou C. X. Ren S. J. (2019). Media or message, which is the king in social commerce?: an empirical study of participants'intention to repost marketing messages on social media. Comput. Hum. Behav. 93, 176–191. 10.1016/j.chb.2018.12.007

[B115] Wübben M. Wangenheim F. V. (2008). Instant customer base analysis: managerial heuristics often “get it right.” J. Market. 72, 82–93. 10.1509/jmkg.72.3.082

[B116] Xiang L. Zheng X. Lee M. K. O. Zhao D. (2016). Exploring consumers'impulse buying behavior on social commerce platform: the role of parasocial interaction. Int. J. Inform. Manage. 36, 333–347. 10.1016/j.ijinfomgt.2015.11.002

[B117] Yang X. Zhang J. Peracchio L. A. (2010). Understanding the impact of self-concept on the stylistic properties of images. J. Consum. Psychol. 20, 508–520. 10.1016/j.jcps.2010.06.012

[B118] Zulli D. (2018). Capitalizing on the look: insights into the glance, attention economy, and Instagram. Crit. Stud. Media Commun. 35, 137–150. 10.1080/15295036.2017.1394582

